# SARS-CoV-2 escape from a highly neutralizing COVID-19 convalescent plasma

**DOI:** 10.1073/pnas.2103154118

**Published:** 2021-08-20

**Authors:** Emanuele Andreano, Giulia Piccini, Danilo Licastro, Lorenzo Casalino, Nicole V. Johnson, Ida Paciello, Simeone Dal Monego, Elisa Pantano, Noemi Manganaro, Alessandro Manenti, Rachele Manna, Elisa Casa, Inesa Hyseni, Linda Benincasa, Emanuele Montomoli, Rommie E. Amaro, Jason S. McLellan, Rino Rappuoli

**Affiliations:** ^a^Monoclonal Antibody Discovery Lab, Fondazione Toscana Life Sciences, 53100 Siena, Italy;; ^b^VisMederi S.r.l., 53100 Siena, Italy;; ^c^ARGO Open Lab Platform for Genome Sequencing, 34149 Trieste, Italy;; ^d^Department of Chemistry and Biochemistry, University of California San Diego, La Jolla, CA 92093;; ^e^Department of Molecular Biosciences, The University of Texas at Austin, Austin, TX 78712;; ^f^VisMederi Research S.r.l., 53100 Siena, Italy;; ^g^Department of Molecular and Developmental Medicine, University of Siena, 53100 Siena, Italy;; ^h^Faculty of Medicine, Imperial College, SW7 2DD London, United Kingdom

**Keywords:** SARS-CoV-2, COVID-19, emerging variants, immune evasion, antibody response

## Abstract

To investigate the evolution of severe acute respiratory syndrome coronavirus 2 (SARS-CoV-2) in the immune population, we coincupi bated the authentic virus with a highly neutralizing plasma from a COVID-19 convalescent patient. The plasma fully neutralized the virus for seven passages, but, after 45 d, the deletion of F140 in the spike N-terminal domain (NTD) N3 loop led to partial breakthrough. At day 73, an E484K substitution in the receptor-binding domain (RBD) occurred, followed, at day 80, by an insertion in the NTD N5 loop containing a new glycan sequon, which generated a variant completely resistant to plasma neutralization. Computational modeling predicts that the deletion and insertion in loops N3 and N5 prevent binding of neutralizing antibodies. The recent emergence in the United Kingdom, South Africa, Brazil, and Japan of natural variants with similar changes suggests that SARS-CoV-2 has the potential to escape an effective immune response and that vaccines and antibodies able to control emerging variants should be developed.

## Significance

This work shows that, under strong immune pressure, SARS-CoV-2 can use mutations in both the N-terminal domain and the receptor-binding domain to escape potent polyclonal neutralizing responses. Indeed, after a long period under immune selective pressure, SARS-CoV-2 evolved to evade the immunity of a potent polyclonal serum from a COVID-19 convalescent donor. Only three mutations were sufficient to generate this escape variant. The new virus was resistant to 70% of the neutralizing antibodies tested and had a decreased susceptibility to all convalescent sera. Our data predict that, as the immunity in the population increases, following infection and vaccination, new variants will emerge, and therefore vaccines and monoclonal antibodies need to be developed to address them.

Severe acute respiratory syndrome coronavirus 2 (SARS-CoV-2), causative agent of COVID-19, accounts for over 105 million cases of infections and more than 2.3 million deaths worldwide. Thanks to an incredible scientific and financial effort, several prophylactic and therapeutic tools, such as vaccines and monoclonal antibodies (mAbs), have been developed in less than 1 y to combat this pandemic (1–4). The main target of vaccines and mAbs is the SARS-CoV-2 spike protein (S protein), a large class I trimeric fusion protein which plays a key role in viral pathogenesis (3, 5, 6). The SARS-CoV-2 S protein is composed of two subunits: S1, which contains the receptor-binding domain (RBD) responsible for the interaction with receptors on the host cells, and S2, which mediates membrane fusion and viral entry (7, 8). The S1 subunit presents two highly immunogenic domains, the N-terminal domain (NTD) and the RBD, which are the major targets of polyclonal and monoclonal neutralizing antibodies (4, 9, 10). The continued spread in immune-competent populations has led to adaptations of the virus to the host and generation of new SARS-CoV-2 variants. Indeed, S-protein variants have been recently described in the United Kingdom, South Africa, Brazil, and Japan (11–13), and the Global Initiative on Sharing All Influenza Data (GISAID) database reports more than 1,100 amino acid changes in the S protein (14, 15).

An important question for vaccine development is whether the authentic virus, under the selective pressure of the polyclonal immune response in convalescent or vaccinated people, can evolve to fully escape immunity and antibody treatment. To address this question, we incubated the authentic SARS-CoV-2 wild-type (WT) virus for more than 90 d in the presence of a potent neutralizing plasma.

## Results

### Characterization of COVID-19 Convalescent Donor Plasma Samples.

Plasma samples from 20 convalescent patients with confirmed COVID-19 infection were collected for this study. All plasmas were collected between March and May 2020 where only the original Wuhan virus and D614G variants were circulating. All plasmas, tested by enzyme-linked immunosorbent assay (ELISA), were found to bind the SARS-CoV-2 S-protein trimer, and most of them also bound the S1 and S2 subunits, and the RBD. However, a broad range of reactivity profiles were noticed, ranging from weak binders with titers of 1/10 to strong binders with titers of 1/10,240 (Table 1 and *SI Appendix*, Fig. S1*A*). PT008, PT009, PT015, PT122, and PT188 showed the strongest binding toward the S trimer, and, among them, PT188 had also the highest binding to the S1–S2 subunits and among the highest binding titers against the RBD (1/1,280). All but one plasma sample (PT103) were able to bind the S-protein S1 subunit, while three plasma samples (PT103, PT200, and PT276) were negative for binding to the RBD. Neutralization activity tested against the SARS-CoV-2 WT and D614G variant also showed variable titers. Most of the plasma samples neutralized the viruses with titers ranging from 1/20 to 1/320. Four samples had extremely low titers (1/10), whereas sample PT188 showed extremely high titers (1/10,240). Four plasma samples did not show neutralization activity against the SARS-CoV-2 WT and SARS-CoV-2 D614G variant. Plasma from subject PT188, which had the highest neutralizing titer and ELISA binding reactivity (Table 1 and *SI Appendix*, Fig. S1 *B–D*), was selected to test whether SARS-CoV-2 can evolve to escape a potent humoral immunity.

**Table 1.** Summary of COVID-19 convalescent plasma characteristics

Sample ID	S-protein trimer-binding titer	RBD-binding titer	S1-binding titer	S2-binding titer	Neutralization titer WT	Neutralization titer D614G	Neutralization titer PT188-EM
PT003	1/320	1/10	1/80	1/320	1/15	Not neutralizing	Not neutralizing
PT004	1/2,560	1/80	1/320	1/2,560	1/120	1/60	1/20
PT005	1/320	1/80	1/160	1/1,280	1/80	1/30	1/10
PT006	1/640	1/160	1/1,280	1/640	1/120	1/20	1/10
PT008	1/10,240	1/80	1/640	1/640	1/120	1/80	1/40
PT009	1/10,240	1/2,560	1/1,280	1/2,560	1/640	1/320	1/120
PT010	1/320	1/80	1/80	1/2,560	1/15	1/10	1/10
PT012	1/1,280	1/160	1/320	1/320	1/120	1/80	1/15
PT014	1/1,280	1/80	1/160	1/1,280	1/120	1/40	1/20
PT015	1/10,240	1/10,240	1/2,560	1/5,120	1/640	1/320	1/160
PT041	1/640	1/40	1/160	1/80	1/40	1/10	1/10
PT042	1/5,120	1/320	1/1,280	1/5,120	1/960	1/320	1/60
PT100	1/1,280	1/80	1/160	1/1,280	1/80	1/30	1/40
PT101	1/640	1/40	1/160	1/320	1/20	1/10	1/10
PT102	1/160	1/20	1/80	1/640	1/10	Not neutralizing	Not neutralizing
PT103	1/160	Not binder	Not binder	1/160	Not neutralizing	Not neutralizing	Not neutralizing
PT122	1/10,240	1/1,280	1/1,280	1/2,560	1/640	1/480	1/320
PT188	1/10,240	1/1,280	1/5,120	1/5,120	1/10,240	1/10,240	1/40
PT200	1/1,280	Not binder	1/160	1/10,240	1/60	1/30	Not neutralizing
PT276	1/80	Not binder	1/80	1/320	Not neutralizing	Not neutralizing	Not neutralizing

The table shows the binding profile and neutralization activities of 20 COVID-19 convalescent plasma samples.

### Evolution of SARS-CoV-2 Convalescent Plasma Escape Mutant.

**Fig. 1.**
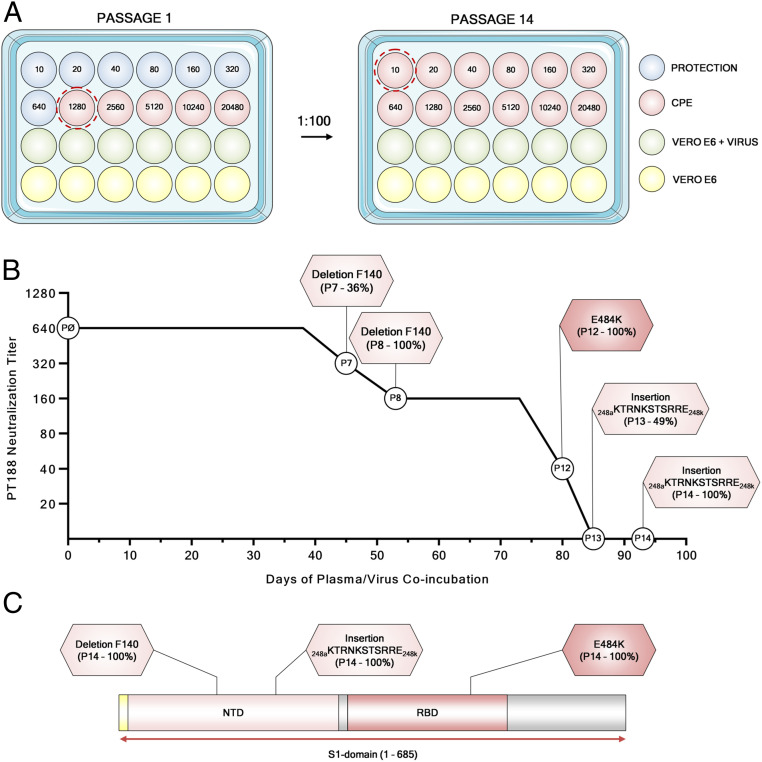
Evolution of an authentic SARS-CoV-2 escape mutant. (*A*) Schematic representation of the 24-well plate format used to select the authentic SARS-CoV-2 escape mutant. Blue, red, green, and yellow wells show feeder cells protect from PT188 neutralization, CPE, authentic virus on Vero E6 cells, and Vero E6 alone, respectively. (*B*) The graph shows the PT188 neutralization titer after each mutation acquired by the authentic virus. Specific mutations, fold decrease, and days on which the mutations occur are reported in the figure. (*C*) SARS-CoV-2 S-protein gene showing type, position of mutations, and frequency of mutations.

Twofold dilutions of plasma PT188 ranging from 1/10 to 1/20,480 were coincubated with 10^5^ median tissue culture infectious dose (TCID_50_) of the WT virus in a 24-well plate. This viral titer was approximately 3 logs more than what is conventionally used in microneutralization assays (16–20). The plasma/virus mixture was coincubated for 5 d to 8 d. Then, the first well showing cytopathic effect (CPE) was diluted 1:100 and incubated again with serial dilutions of plasma PT188 (Fig. 1*A* and *SI Appendix*, Table S1). For six passages and 38 d, PT188 plasma neutralized the virus with a titer of 1/640 and did not show any sign of escape. However, after seven passages and 45 d, the neutralizing titer decreased to 1/320. Sequence analyses revealed a deletion of the phenylalanine in position 140 (F140) on the S-protein NTD N3 loop in 36% of the virions (Fig. 1 *B* and *C* and *SI Appendix*, Table S1). In the subsequent passage (P8), this mutation was observed in 100% of the sequenced virions, and an additional twofold decrease in neutralization activity was observed, reaching an overall neutralization titer of 1/160. Following this initial breakthrough, a second mutation occurred after 12 passages and 80 d of plasma/virus coincubation (P12). This time, the glutamic acid in position 484 of the RBD was substituted with a lysine (E484K). This mutation occurred in 100% of sequenced virions and led to a fourfold decrease in neutralization activity which reached a titer of 1/40 (Fig. 1 *B* and *C* and *SI Appendix*, Table S1). The E484K substitution was rapidly followed by a third and final change comprising an 11-amino acid insertion between Y248 and L249 in the NTD N5 loop (_248a_KTRNKSTSRRE_248k_). The insertion contained an N-linked glycan sequon (_248d_NKS_248f_), and this viral variant resulted in complete abrogation of neutralization activity by the PT188 plasma sample. Initially, this insertion was observed in only 49% of the virions, but, when the virus was kept in culture for another passage (P14), the insertion was fully acquired by the virus (Fig. 1 *B* and *C* and *SI Appendix*, Table S1).

### Reduced Susceptibility to Convalescent Plasma and Monoclonal Antibodies.

**Fig. 2.**
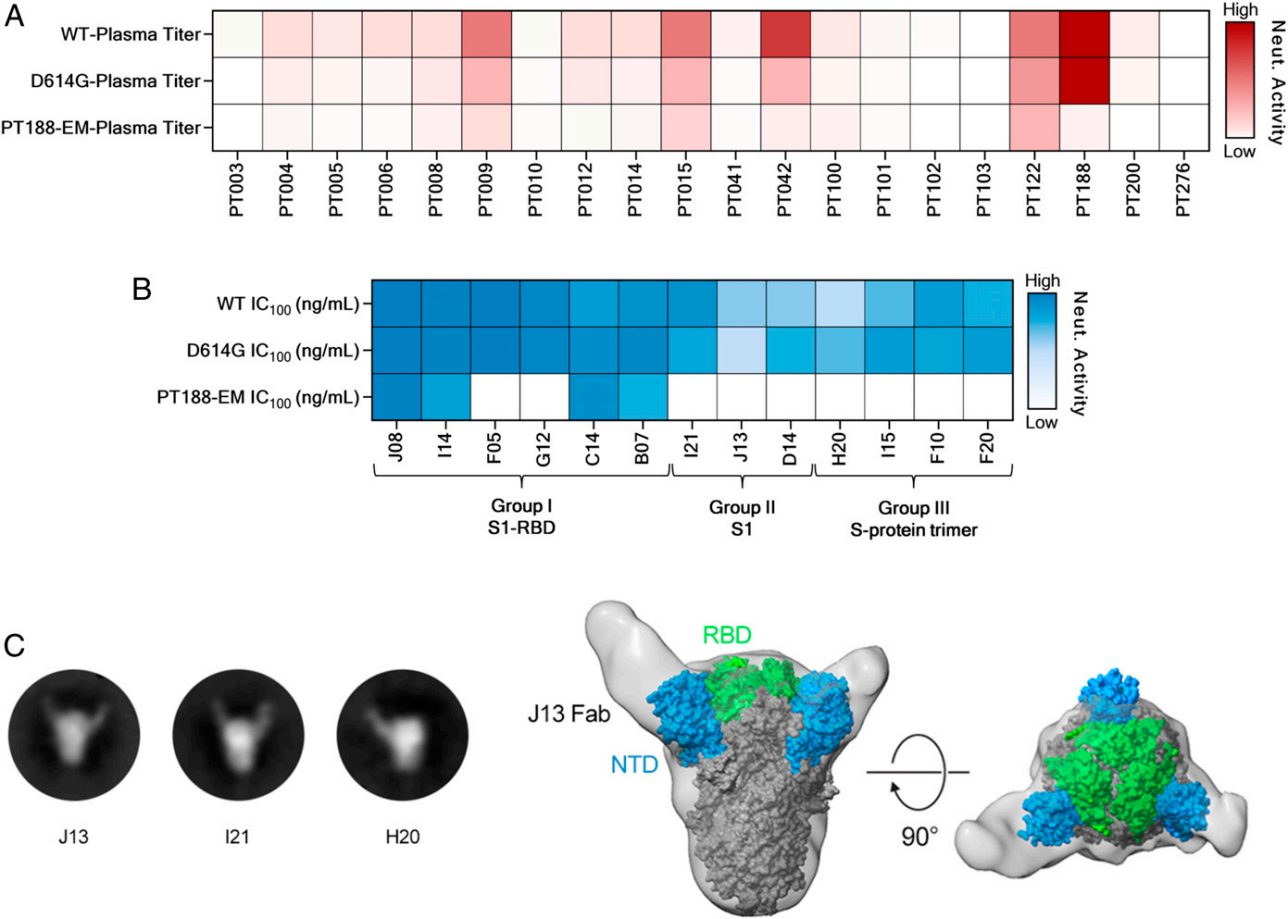
Neutralization (Neut.) efficacy of plasma and 13 mAbs against SARS-CoV-2 PT188-EM. (*A*) Heat map showing the neutralization activity of tested plasma samples to the SARS-CoV-2 WT and D614G and PT188-EM variants. (*B*) Heat maps showing neutralization profiles of tested mAbs. (*C*) Negative stain EM 2D class averages showing J13, I21, and H20 Fabs bound to the SARS-CoV-2 S protein. (*D*) A 3D reconstruction of J13 bound to the NTD domain of the S protein viewed looking along (*Left*) or toward (*Right*) the viral membrane.

To evaluate the ability of the SARS-CoV-2 PT188 escape mutant (PT188-EM) to evade the polyclonal antibody response, all 20 plasma samples from COVID-19 convalescent patients were tested in a traditional CPE-based neutralization assay against this viral variant using the virus at 100 TCID_50_. All samples showed at least a twofold decrease in neutralization activity against SARS-CoV-2 PT188-EM (Fig. 2*A*, Table 1, and *SI Appendix*, Fig. S1 *B–D*). As expected, the plasma used to select the escape mutant showed the biggest neutralization decrease against this escape mutant with a 256-fold decrease compared to WT SARS-CoV-2. Plasma PT042, PT006, PT005, PT012, and PT041 also showed a substantial drop in neutralization efficacy (Table 1). In addition, we observed that a higher response toward the S-protein S1 subunit correlates with loss of neutralization activity against SARS-CoV-2 PT188-EM (see *SI Appendix*, Fig. S2*A*), whereas a high response toward the S-protein S2 subunit did not show correlation (see *SI Appendix*, Fig. S2*B*).

We also tested a previously identified panel of 13 neutralizing mAbs (nAbs) by CPE-based neutralization assay to assess their neutralization efficacy against SARS-CoV-2 PT188-EM. These antibodies were classified into three groups based on their binding profiles to the S protein. Group I nAbs were able to bind the S1-RBD, group II targeted the S1 subunit but not the RBD, and group III nAbs were specific for the S-protein trimer (Table 2). These antibodies also showed a variable neutralization potency against the SARS-CoV-2 WT and D614G viruses ranging from 3.9 ng/mL to 500.0 ng/mL (Fig. 2*B*, Table 2, and *SI Appendix*, Fig. S1 *E–G*). The three mutations selected by SARS-CoV-2 PT188-EM to escape the highly neutralizing plasma completely abrogated the neutralization activity of two of the six tested RBD-directed antibodies (F05 and G12) (Fig. 2*B*, Table 2, and *SI Appendix*, Fig. S1 *E–G*), suggesting that their epitopes include E484. In contrast, the extremely potent neutralizing antibody J08 was the most potently neutralizing antibody against this escape mutant, with an IC_100_ of 22.1 ng/mL. Interestingly, the S1-RBD−directed antibody C14 showed a twofold increase in neutralization activity compared to the SARS-CoV-2 WT virus, whereas I14 and B07 showed a 16-fold and twofold decrease, respectively. All tested antibodies derived from group II (S1-specific not RBD) and group III (S-protein trimer specific) completely lost their neutralization ability against SARS-CoV-2 PT188-EM (Fig. 2*B*, Table 2, and *SI Appendix*, Fig. S1 *E–G*). To better understand the abrogation of activity of some of the tested antibodies, J13, I21, and H20 were cocomplexed with SARS-CoV-2 WT S protein and structurally evaluated by negative-stain EM. Two-dimensional (2D) class averages of the three tested antibodies showed that they all bind to the NTD of the S protein (Fig. 2*C*). A 3D reconstruction for the J13 Fab complex provided further evidence that this antibody binds to the NTD (Fig. 2*D*).

**Table 2.** Features of 13 SARS-CoV-2 neutralizing antibodies

mAb ID	Binding specificity*	Neutralization WT IC_100_ (ng:mL)*	Neutralization D614G IC_100_ (ng:mL)*	Neutralization PT188-EM IC_100_ (ng:mL)
J08	S1-RBD	3.9	7.8	22.1
I14	S1-RBD	11.0	19.7	176.8
F05	S1-RBD	3.9	4.9	Not neutralizing
G12	S1-RBD	39.4	39.4	Not neutralizing
C14	S1-RBD	157.5	78.7	88.4
B07	S1-RBD	99.2	49.6	250.0
I21	S1	99.2	198.4	Not neutralizing
J13	S1	396.8	500.0	Not neutralizing
D14	S1	396.8	250.0	Not neutralizing
H20	S protein	492.2	310.0	Not neutralizing
I15	S protein	310.0	155.0	Not neutralizing
F10	S protein	155.0	195.3	Not neutralizing
F20	S protein	246.1	155.0	Not neutralizing

The table shows the binding and neutralization profile of 13 previously identified SARS-CoV-2 nAbs.

* Column refers to previously published data (1).

### Putative Structural Effects Enabling Viral Escape.

**Fig. 3.**
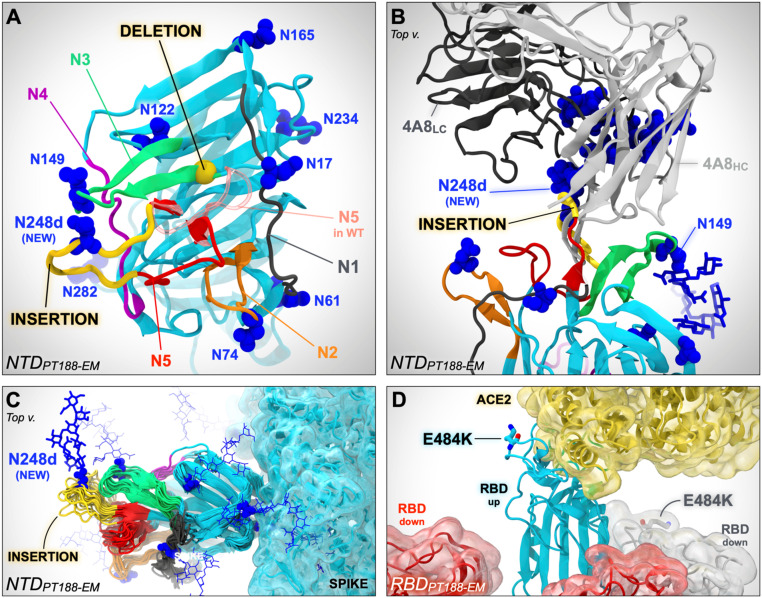
In silico modeling of the PT188-EM spike NTD and RBD. (*A*) In silico model of the NTD of the SARS-CoV-2 PT188-EM spike protein based on PDB ID code 7JJI. This model accounts for the 11-amino acid insertion (yellow ribbon) and F140 deletion (highlighted with a yellow bead). N5 loop as in the WT cryo-EM structure (PDB ID code 7JJI) is shown as a transparent red ribbon. (*B*) Close-up of the PT188-EM spike NTD model in complex with antibody 4A8. Both heavy chain (HC, light gray) and light chain (LC, dark gray) of 4A8 are shown. The 11-amino acid insertion (yellow ribbon) within N5 loop introduces a new N-linked glycan (N248d) that sterically clashes with 4A8, therefore disrupting the binding interface. The N-glycan at position N149 is, however, compatible with 4A8 binding. (*C*) Conformational dynamics of the PT188-EM spike NTD model resulting from 100 ns of MD simulation is shown by overlaying multiple frames along the generated trajectory. (*D*) In silico model of the PT188-EM spike RBD based on PDB ID code 6M17, where the E484K mutation is shown with licorice representation.

Computational modeling and simulation of the WT and PT188-EM spikes provides a putative structural basis for understanding antibody escape. The highly antigenic NTD is more extensively mutated, containing the F140 deletion as well as the 11-amino acid insertion in loop N5 that introduces a novel N-glycan sequon at position N248d (Fig. 3 *A–C*). In contrast, the single mutation in the RBD (E484K) swaps the charge of the sidechain, which would significantly alter the electrostatic complementarity of antibody binding to this region (Fig. 3*D*). Upon inspection of molecular dynamics (MD) simulations of the NTD escape mutant model, we hypothesize that the F140 deletion alters the packing of the N1, N3, and N5 loops (see *SI Appendix*, Fig. S3), where the loss of the bulky aromatic sidechain would overall reduce the stability of this region (Table 1). Subsequently, the extensive insertion within the N5 loop appears to remodel this critical antigenic region, predicting substantial steric occlusion with antibodies targeting this epitope, such as antibody 4A8 (Fig. 3*B*) (21). Furthermore, introduction of a new N-glycan at position N248d (mutant numbering scheme) would effectively eliminate neutralization by such antibodies (Fig. 3*B* and *SI Appendix*, Fig. S4).

### Escape Mutant Shows Similar Viral Fitness Compared to the WT Virus.

To determine the extent to which the escape mutations were detrimental to the infectivity of SARS-CoV-2 PT188-EM, the viral fitness was evaluated. Four different measures were assessed: visible CPE, viral titer, RNA-dependent RNA polymerase (RdRp), and nucleocapsid (N) RNA detection by RT-PCR (see *SI Appendix*, Fig. S5). Initially, the SARS-CoV-2 WT virus and the PT188-EM variant were inoculated at a multiplicity of infection of 0.001 on Vero E6 cells. Every day, for four consecutive days, a titration plate was prepared and optically assessed after 72 h of incubation to evaluate the CPE effect on Vero E6 cells and viral titer. Furthermore, the RNA was extracted to assess RdRp and N-gene levels in the supernatant. We collected pictures at 72 h postinfection to evaluate the morphological status of noninfected Vero E6 cells and the CPE on infected feeder cells. Vero E6 cells were confluent at 72 h, and no sign of CPE was optically detectable (see *SI Appendix*, Fig. S5*A*). Conversely, SARS-CoV-2 WT and PT188-EM showed significant and comparable amounts of CPE (see *SI Appendix*, Fig. S5*A*). Viral titers were evaluated for both SARS-CoV-2 WT and PT188-EM, and no significant differences were observed, as the viruses showed almost identical growth curves (see *SI Appendix*, Fig. S5*B*). A similar trend was observed when RdRp and N-gene levels in the supernatant were detected, even if slightly higher levels of RdRp and N gene were detectable for SARS-CoV-2 PT188-EM at day 0 and day 1 (see *SI Appendix*, Fig. S5*C*). Finally, strong correlations between viral titers and RdRp/N-gene levels were observed for both SARS-CoV-2 WT and PT188-EM (see *SI Appendix*, Fig. S5 *D* and *E*).

## Discussion

We have shown that the authentic SARS-CoV-2, if constantly pressured, has the ability to escape even a potent polyclonal serum targeting multiple neutralizing epitopes. These results are remarkable because SARS-CoV-2 shows a very low estimated evolutionary rate of mutation, as this virus encodes a proofreading exoribonuclease machinery, and, therefore, while escape mutants can be easily isolated when viruses are incubated with single mAbs, it is usually believed that a combination of two mAbs is sufficient to eliminate the evolution of escape variants (22–25). The recent isolation of SARS-CoV-2 variants in the United Kingdom, South Africa, Brazil, and Japan with deletions in or near the NTD loops shows that what we describe here can occur in the real world. The ability of the virus to adapt to the host immune system was also observed in clinical settings where an immunocompromised COVID-19 patient, after 154 d of infection, presented different variants of the virus, including the E484K substitution (26). Therefore, we should be prepared to deal with virus variants that may be selected by the immunity acquired from infection or vaccination. This can be achieved by developing second-generation vaccines and mAbs, possibly targeting universal epitopes and able to neutralize emerging variants of the virus.

A limitation of this study is that viral evolution of SARS-CoV-2 was evaluated only for one plasma sample, limiting the observation of possible spike protein mutations only to a specific polyclonal response. In fact, PT188-EM impacted our plasma samples differently, where PT188, used to pressure the virus in vitro, was the most impacted sample (256-fold decrease), while the remaining 15 neutralizing plasmas showed a median neutralization titer reduction of ∼sevenfold.

Our data also confirm that the SARS-CoV-2 neutralizing antibodies acquired during infection target almost entirely the NTD and the RBD. In the RBD, the possibility to escape is limited, and the mutation E484K that we found is one of the most frequent mutations to escape mAbs (22) and among the most common RBD mutations described in experimental settings (27). Remarkably, the evolution of the E484K substitution observed in our experimental setting was replicated a few months later in the real world by the emergence of E484K variants in South Africa, Brazil, and Japan (14). This is likely due to residue E484 being targeted by antibodies derived from IGHV3-53 and closely related IGHV3-66 genes, which are the most common germlines for antibodies directed against the RBD (28). Recently, this mutation has also been shown to reduce considerably the neutralizing potency of vaccine-induced immunity and to escape mAbs already approved for emergency use by the Food and Drug Administration (29–31).

On the other hand, the NTD loops can accommodate many different changes, such as insertions, deletions, and amino acid alterations. Interestingly, in our case, the final mutation contained an insertion carrying an N-glycosylation site which has the potential to hide or obstruct the binding to neutralizing epitopes. The introduction of a glycan is a well-known immunogenic escape strategy described in influenza (32), HIV-1, and other viruses (33–35), although this finding presents a patient-derived escape mutant utilizing this mechanism for SARS-CoV-2. Surprisingly, only three mutations, which led to complete rearrangement of NTD N3 and N5 loops and substitution to a key residue on the RBD, were sufficient to eliminate the neutralization ability of a potent polyclonal serum. Fortunately, not all plasma and mAbs tested were equally affected by the three mutations, suggesting that natural immunity to infection can target additional epitopes that can still neutralize the PT188-EM variant. Vaccine-induced immunity, which is more robust than natural immunity, is likely to be less susceptible to emerging variants. Indeed, so far, the virus has not mutated sufficiently to completely avoid the antibody response raised by current vaccines (36, 37).

Going forward, it will be important to continue to closely monitor which epitopes on the S protein are targeted by the vaccines against SARS-CoV-2 that are being deployed in hundreds of millions of people around the world.

## Materials and Methods

### Enrollment of SARS-CoV-2 Convalescent Donors and Human Sample Collection.

COVID-19 convalescent plasma samples were provided by the National Institute for Infectious Diseases, Institute for Scientific Based Recovery and Cure—Lazzaro Spallanzani Rome (Italy) and Azienda Ospedaliera Universitaria Senese, Siena (Italy). Samples were collected from convalescent donors who gave their written consent. The study was approved by local ethics committees (Parere 18_2020 in Rome and Parere 17065 in Siena) and conducted according to good clinical practice in accordance with the Declaration of Helsinki (European Council 2001, US Code of Federal Regulations, International Conference on Harmonization 1997). This study was unblinded and not randomized.

### SARS-CoV-2 Authentic Virus Neutralization Assay.

The mAbs and plasma neutralization activity was evaluated using a CPE-based assay as previously described (17, 20). Further details are available in *SI Appendix*, *Materials and Methods*.

### Viral Escape Assay Using Authentic SARS-CoV-2.

All SARS-CoV-2 authentic virus procedures were performed in the biosafety level 3 (BSL3) laboratories at Toscana Life Sciences in Siena (Italy) and Vismederi S.r.l., Siena (Italy). BSL3 laboratories are approved by a certified biosafety professional and are inspected every year by local authorities. To detect neutralization-resistant SARS-CoV-2 escape variants, a standard concentration of the virus was sequentially passaged in cell cultures in the presence of serially diluted samples containing SARS-CoV-2-specific antibodies. Briefly, 12 serial twofold dilutions of PT188 plasma prepared in complete Dulbecco’s modified Eagle’s medium 2% fetal bovine serum (starting dilution 1:10) were added to the wells of one 24-well plate. Virus solution containing 10^5^ TCID_50_ of authentic SARS-CoV-2 was dispensed in each antibody-containing well, and the plates were incubated for 1 h at 37 °C, 5% CO_2_. The mixture was then added to the wells of a 24-well plate containing a subconfluent Vero E6 cell monolayer. Plates were incubated for 5 d to 7 d at 37 °C, 5% CO_2_ and examined for the presence of CPE using an inverted optical microscope. A virus-only control and a cell-only control were included in each plate to assist in distinguishing absence or presence of CPE. At each virus passage, the content of the well corresponding to the lowest sample dilution that showed complete CPE was diluted 1:100 and transferred to the antibody-containing wells of the predilution 24-well plate prepared for the subsequent virus passage. At each passage, both the virus pressured with PT188 and the virus-only control were harvested, propagated in 25-cm^2^ flasks, and aliquoted at −80 °C to be used for RNA extraction, RT-PCR, and sequencing.

### Negative Stain Electron Microscopy.

SARS-CoV-2 S protein was expressed and purified as previously described (38). Purified spike was combined with individual Fabs at final concentrations of 0.04 mg/mL and 0.16 mg/mL, respectively. Following a 30-min incubation on ice, each complex was deposited on plasma cleaned CF-400 grids (EMS) and stained using methylamine tungstate (Nanoprobes). Grids were imaged at 92,000× magnification in a Talos F200C transmission electron microscope (TEM) equipped with a Ceta 16M detector (Thermo Fisher Scientific). Contrast transfer function estimation and particle picking were performed using cisTEM (39), and particle stacks were exported to cryoSPARC v2 (40) for 2D classification, ab initio 3D reconstruction, and heterogeneous refinement.

### Computational Methods.

The PT188-EM spike escape mutant was modeled using in silico approaches. As the mutations are localized in two different domains of the spike, namely the NTD and the RBD, separate models were generated for each domain. In detail, two models of the PT188-EM spike NTD (residues 13 to 308) were built starting from two different cryoelectron microscopy (cryo-EM) structures of the WT S protein as templates: 1) one bearing a completely resolved NTD [Protein Data Bank (PDB) ID code 7JJI (41)], which includes all the loops from N1 to N5, and 2) one bound to the antibody 4A8 [PDB ID code 7C2L (21)], which presents only one small gap within the N5 loop. The model of the PT188-EM spike RBD was based on the cryo-EM structure of the spike’s RBD in complex with ACE2 [PDB ID code 6M17 (42)]. The generated models were subsequently refined using explicitly solvated all-atom MD simulations. The systems and the simulations were visually inspected with visual molecular dynamics, which was also used for image rendering (43). Further details on the computational method analyses are reported in *SI Appendix*, *Materials and Methods*.

## Supplementary Material

Supplementary File

## Data Availability

All study data are included in the article and *SI Appendix*.
