# Supplementary material for: SARS-CoV-2 escape from a highly neutralizing COVID-19 convalescent plasma
Source: Proc Natl Acad Sci U S A. 2021 Aug 20;118(36):e2103154118. doi: 10.1073/pnas.2103154118 (PMC8433494; doi:10.1073/pnas.2103154118)
Supplement: Supplementary File [file pnas.2103154118.sapp.pdf]

PNAS
www.pnas.org

Supplementary Information for

SARS-CoV-2 escape from a highly neutralizing COVID-19 convalescent plasma

Emanuele Andreano, Giulia Piccini, Danilo Licastro, Lorenzo Casalino, Nicole V. Johnson, Ida Paciello, Simeone Dal Monego, Elisa Pantano, Noemi Manganaro, Alessandro Manenti, Rachele Manna, Elisa Casa, Inesa Hyseni, Linda Benincasa, Emanuele Montomoli, Rommie E. Amaro, Jason S. McLellan, Rino Rappuoli*

* To whom correspondence should be addressed: Dr. Rino Rappuoli

Email: rino.r.rappuoli@gsk.com

This PDF file includes:

Material and Methods
Figure S1
Figure S2
Figure S3
Figure S4
Figure S5
Table S1
SI References

Material and Methods

ELISA assay with SARS-CoV-2 S-protein prefusion trimer, S1 – S2 subunits and RBD

COVID-19 convalescent plasmas were screened by ELISA to profile their binding to the SARS-CoV-2 S-protein, S1 - S2 subunits, and RBD (1). Briefly, 384-well plates were coated with 3 µg/mL of streptavidin diluted in coating buffer (0.05 M carbonate-bicarbonate solution, pH 9.6) and incubated at room temperature (RT) overnight. Plates were then coated with SARS-CoV-2 S-protein, S1 or S2 subunits or RBD at 3 µg/mL and incubated for 1h at RT. 50 µL/well of saturation buffer (PBS with 1% BSA) was used to saturate unspecific binding and plates were incubated at 37°C for 1h without CO_2. Plasma samples were diluted 1:10 in PBS/BSA 1%/Tween20 0.05% in 25 µL/well final volume and incubated for 1h at 37°C without CO_2. Following, 25 µL/well of alkaline phosphatase-conjugated goat anti-human IgG (Sigma-Aldrich) was used as secondary antibodies. Wells were washed three times between each step with PBS/BSA 1%/Tween20 0.05%. Finally, pNPP (p-nitrophenyl phosphate) (Sigma-Aldrich) was used as soluble substrate to detect the polyclonal response to SARS-CoV-2 S-protein, S1 or S2 subunit or RBD and the final reaction was measured using the Varioskan Lux Reader (Thermo Fisher Scientific) at a wavelength of 405 nm. Samples were considered as positive if OD at 405 nm (OD_{405}) was twice the blank.

Cell culture conditions

African green monkey kidney Vero E6 cells (American Type Culture Collection [ATCC] #CRL-1586) were grown in Dulbecco's Modified Eagle's Medium (DMEM) high glucose supplemented with 2 mM L-glutamine, 100 U/mL of penicillin, 100 µg/mL streptomycin ("complete DMEM" medium) and 10% fetal bovine serum (FBS). Cells were cultured at 37°C, 5% CO_2 and passaged every 3-4 days. 18-24 hours before execution of the viral escape assay, plates and propagation flasks containing a standard concentration of Vero E6 cells were prepared in complete DMEM medium supplemented with 2% FBS and incubated at 37°C, 5% CO_2 until use. 24-well plates were inoculated with $2x10^4$ cells/well to passage the virus-antibody mixture and a virus-only control. 25 cm^2 flasks pre-seeded with $1x10^5$ cells/mL were prepared in parallel to propagate the viral strains of each experiment to obtain a suitable virus concentration for RNA extraction and subsequent sequencing or RT-PCR analysis. 96 well-plates were inoculated with $1.5x10^4$ cells/well and used for titration of the virus-antibody mixture at each passage.

Virus propagation and titration

Wild-type SARS CoV-2 2019 (2019-nCoV strain 2019-nCov/Italy-INMI1) and D614G (SARS-CoV-2/human/ITA/INMI4/2020) viruses were purchased from the European Virus Archive goes Global (EVAg, Spallanzani Institute, Rome). For virus propagation, 175 cm^2 flasks were seeded with Vero E6 cells diluted in complete DMEM high glucose supplemented with 2% FBS at a

concentration of 1x10^{6} cells/mL, and incubated at 37°C, 5% CO_2 for 18-20 hours. After 2 washes with sterile Dulbecco's phosphate-buffered saline (DPBS), the sub-confluent cell monolayer was inoculated with the SARS-CoV-2 virus at a multiplicity of infection (MOI) of 0.001, incubated for 1 hour at 37°C, 5% CO_2. Flasks were then filled with 50 mL of complete DMEM 2% FBS, incubated at 37°C, 5% CO_2, and checked daily until approximately 80-90% of the cell culture showed cytopathic effect (CPE). Supernatants of the infected culture were collected and centrifuged at 4°C, 1,600 rpm for 8 minutes to remove cell debris, aliquoted and stored at -80°C. A titration of the propagated viral stocks was performed in 96-well plates containing confluent Vero E6 monolayers, using a 50% tissue culture infectious dose assay ($TCID_{50}$). Cells infected with serial 10-fold dilutions (10^{-1} to 10^{-11}) of the virus were incubated at 37°C, 5% CO_2 and monitored for signs of virus-induced CPE under an inverted optical microscope for 3–4 days. The viral titer, defined as the reciprocal of the highest viral dilution resulting in at least 50% CPE in the inoculated wells, was calculated with the Spearman-Karber formula (2).

SARS-CoV-2 authentic virus neutralization assay

mAbs were tested at a starting concentration of 1 µg/mL diluted in steps of 1:2 while plasma samples were tested at a starting dilution of 1:10 and then diluted in steps of 1:2 for twelve points. All samples were mixed with a SARS-CoV-2 WT, SARS-CoV-2 D614G or SARS-CoV-2 PT188-EM viral solution containing 100 $TCID_{50}$ of the virus. After 1 hour incubation at 37°C, 5% CO_2, virus-mAb mixture was added to the wells of a 96-well plate containing a sub-confluent Vero E6 cell monolayer. Plates were incubated for 3 days at 37°C in a humidified environment with 5% CO_2, then examined for CPE by means of an inverted optical microscope. Absence or presence of CPE was defined by comparison of each well with the positive control (plasma sample showing high neutralizing activity of SARS-CoV-2 in infected Vero E6 cells) and negative control (human serum sample negative for SARS-CoV-2 in ELISA and neutralization assays and Vero E6 alone). Technical triplicates were performed for each experiment.

RNA Extraction

To isolate the viral genetic material for NGS and detection by RT-qPCR, an RNA extraction step was performed using CommaXP® virus DNA/RNA extraction (Spin Column) commercial kit, according to manufacturer's instruction. Briefly, 500 µL of "Buffer GLX" was added to 300 µL of sample, vortexed for 1 minute and incubated at RT for 5 minutes to allow virus inactivation. The mixture was then added into a spin column inserted in a collection tube and centrifuged at 12,000 rpm for 1 minute at RT. The eluted solution was discarded and 500 µL of "Buffer PD" previously re-suspended in isopropanol were added to the column, then centrifuged as before. Following elimination of the eluted solution, the column was washed with 700 µL of "Buffer PW" previously re-suspended in absolute ethanol and centrifuged as before. This wash was repeated twice. The

spin column was then centrifuged at 12,000 rpm for 2 minutes and left with open lid for 5 minutes to allow evaporation of residual ethanol. The column was placed in a new collection tube and 100 µL of RNAse-free ddH_20 were added. After a 2 minute incubation at RT, the column was centrifuged for 2 minutes at 12,000 rpm to elute the RNA, which was collected in a new tube for PCR analysis.

Library preparation and sequencing

Each viral RNA was retrotranscribed using Superscript IV First-Strand Synthesis System (Thermo Fisher Scientific) without the optional RNase H step and used as input for sequencing library construction. Library preparation was performed with the swift amplicon SARS-CoV-2 research panel (Swift Biosciences, Ann Arbor, MI USA) according to manufacturer's instructions. Library preparation workflow requires two sequential PCR steps. First, more than 341 specific regions are selectively amplified in a targeted single multiplex PCR amplification reaction. Next, a universal PCR is performed to label all amplicons with unique combinations of dual indexed adapters, enabling multiplexing of samples in the same run. Bead-based clean-ups were used to purify the sample by removing unused oligonucleotides and changing buffer composition between steps. Purified individually tagged libraries were quantified by qPCR using Kapa Lib Quant Kit (Roche Diagnostics). In conjunction with the qPCR Ct values we used a library size of 265 bp to calculate library molarity. All the obtained libraries passed quality check and were quantified before being pooled at equimolar concentration and sequenced on Illumina MiSEQ 2x250bp paired-end mode following standard procedures including “Adapter Trimming" and "Adapter Trimming Read 2" option. Sequenced reads were quality trimmed using Trimmomatic software during data analysis. Only good quality reads were mapped against SARS-CoV-2_human_ITA_INMI1_2020 GenBank: MT066156.1 using BWA software with default parameters. After inspection using IGV software, consensus sequences were created for each processed sample.

RT-PCR

Multiplex real time RT-PCR for simultaneous detection of SARS-CoV-2-2019 N gene and RdRp gene was performed using NeoplexTM COVID-19 Detection Kit. The primer and probe system of the kit is based on the standard TaqMan® Technology. SARS-CoV-2 specific probes are labelled with the FAM and JOE fluorophore to target COVID-19 RdRp and N genes, respectively. The internal PCR control contains primers for targeting human RNaseP mRNA and probes labelled with the Cy5 fluorophore. For RT-PCR, 5 µL of extracted RNA, 5 µL of DW/RNase-free water, 5 µL of COVID-19 PPM (containing primers and probes for targeting RdRp gene, N gene, and human RNase mRNA as an internal control) and 5 µL of One-step Master Mix were used in a final reaction volume of 20 µL to be run in a LightCycler® 96 System (Roche). A negative control

consisting of RNase-free water, and a COVID-19 Positive Control (which includes RdRp, N, and internal control target genes as in vitro transcript (IVT) RNA) were included in each run. The PCR cycling conditions used were as follows: reverse transcription was performed at 53°C for 2 minutes, then an initial amplification was done with a denaturation step at 95°C for 2 min, followed by 40 cycles of denaturation at 95°C for 3 sec and primer annealing/extension at 60°C for 30 s. Reactions were run in duplicate in the same experiment. Data were collected by the LightCycler software during the annealing phase of each cycle of amplification. For each sample, a cycle threshold (Ct) was generated for each target (N gene, RdRp and internal control), based on the cycle number where the instrument software detected a log increase in fluorescence of the given sample.

Computational Methods

The 11-amino-acid insertion between Y248 and L249 within the NTD was modeled as a loop using Modeller9.19 (3) and keeping all the original cryo-EM coordinates fixed but allowing a certain extent of flexibility for the flanking residues, namely Y248 and 249-257. The F140 deletion was modeled at the same time as the insertion, only allowing residues 138-141 to be flexible. For both the two PT188-EM spike NTD constructs (i.e., one based on 7JJI and one upon 7C2L), 500 models were independently generated, and the system with the best (i.e., the lowest value) Z-DOPE (Discrete Optimized Protein Energy) score was selected upon visual inspection. The modeled PT188-EM spike NTDs were fully glycosylated at the native N-linked glycosylation sites (N17, N61, N74, N122, N149, N165, N234, N282) and at the new N-linked sequon N248d-K248e-S248f introduced within the 11-amino-acid insertion. The glycosylation profile was chosen to be consistent with available glycoanalytic data (4, 5) and analogously to Casalino et al (6). Although there is no information available for the new glycan at position N248d, this was modeled as FA2 complex-type, similarly to the glycan at position N149 (FA3 complex-type). Starting conformations for the glycans were derived from Casalino et al. (6). For the PT188-EM spike RBD carrying E484K mutation we used a model of the fully glycosylated spike in complex with the human ACE2 receptor previously built by Casalino et al. (7) and based on the RBD/ACE2 complex simulated by Barros et al. (8), which in turn was modelled upon the cryo-EM structure by Yan et al. (9) Using this construct, E484 was mutated into a lysine using PSFGEN within VMD (10).

The two glycosylated constructs for the PT188-EM spike NTD and the one accounting for the E484K mutation within the RBD described above were embedded into an orthorhombic box of explicit waters with a 150 mM concentration of Na+ and Cl- ions, leading to a final size of (i) ~185,151, (ii) 190,246 and (iii) ~1,178,601 atoms, respectively. Protonation states were assessed using PROPKA3 (11) at pH 7.4. The final set up was done with PSFGEN and VMD (10), whereas MD simulations were run on TACC Frontera computer facility using NAMD 2.14 (12) and CHARMM36m force fields to refine the models (13-15). All the simulations were carried out using

a 2 fs timestep with SHAKE (16) algorithm to keep the bonds involving hydrogen atoms fixed. The cutoff for non-bonded van der Waals and short-range electrostatic interactions was set to 12 Å, whereas the particle-mesh Ewald (17) approach was employed to account for long-range electrostatics. All three systems were first minimized using the conjugate gradient energy approach for 10,000 steps. Subsequently the temperature of the systems was gradually increased to 310 K for 1 ns in NVT ensemble, while imposing a harmonic restraint of 5 kcal/mol to the protein and the glycan atoms. Next, the restraints were released, and the systems were coupled to a Langevin thermostat (18) (310 K) and a Nosé-Hoover Langevin piston (19, 20) (1.01325 bar) and equilibrated for ~5 ns in NPT conditions. After this point only the PT188-EM spike NTD model (i) based on 7JJI was subjected to production run MD for ~100 ns to check for the flexibility of the 11 aa insertion loop and the impact of the F140 deletion, whereas the simulations of the other PT188-EM spike NTD model (ii) based on 7CL2 and of the PT188-EM spike RBD model were stopped.

Supplementary Figures

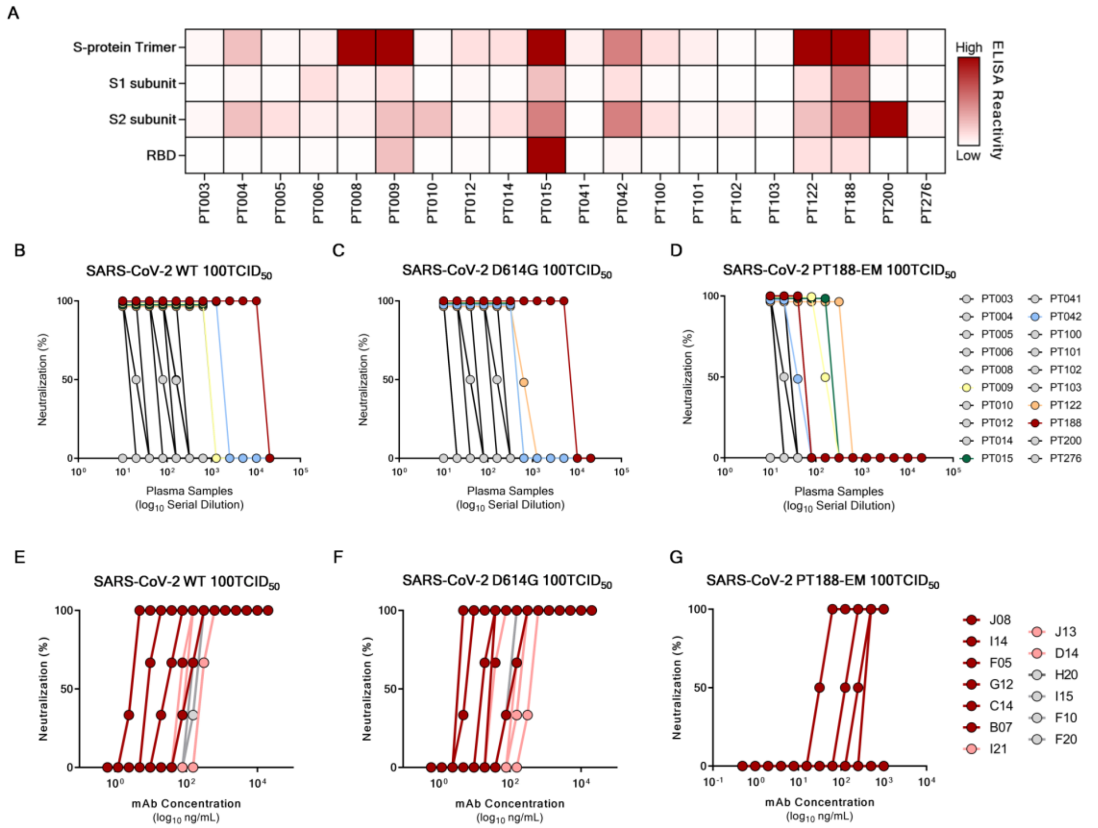

Figure S1. Binding and neutralization profiling of plasma samples from twenty COVID-19 convalescent patients. (A) Binding profiling of COVID-19 convalescent plasma to S-protein trimer, S1 subunit, S2 subunit and RBD. (B – D) Neutralization activity of COVID-19 convalescent plasma against SARS-CoV-2 WT (B) and D614G (C) viruses and SARS-CoV-2 PT188-EM (D). Data are representative of technical triplicates. All plasma are shown in grey, only most potently neutralizing plasma were colored in yellow, green, light blue, orange and red for PT009, PT015, PT042, PT122 and PT188 respectively. (E – G) Neutralization curves for the thirteen tested mAbs against SARS-CoV-2 WT (E), SARS-CoV-2 D614G (F) and SARS-CoV-2 PT188-EM respectively (G). Data are representative of technical triplicates.

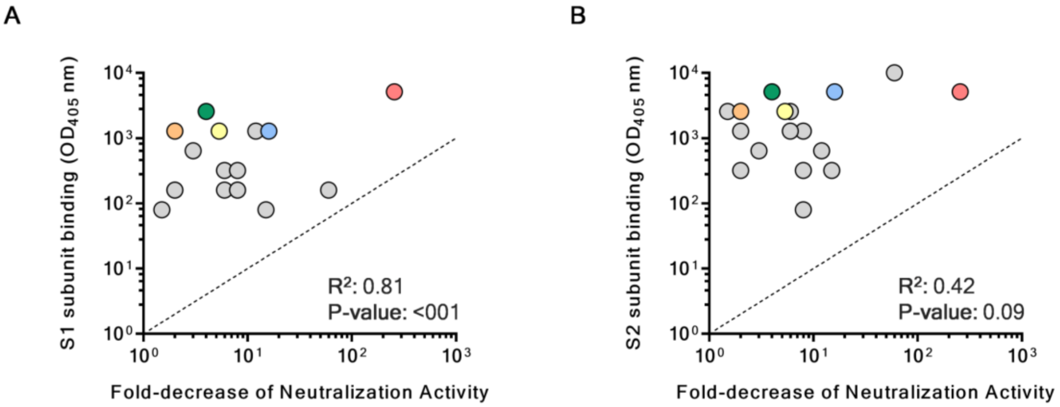

Figure S2. Binding and functional characterization of COVID-19 convalescent plasma. (A – B) Graphs show correlation between S1 subunit (A) and S2 subunit (B) binding and fold-decrease of neutralization activity. All plasma are shown in grey, only most potently neutralizing plasma were colored in yellow, green, light blue, orange and red for PT009, PT015, PT042, PT122 and PT188 respectively.

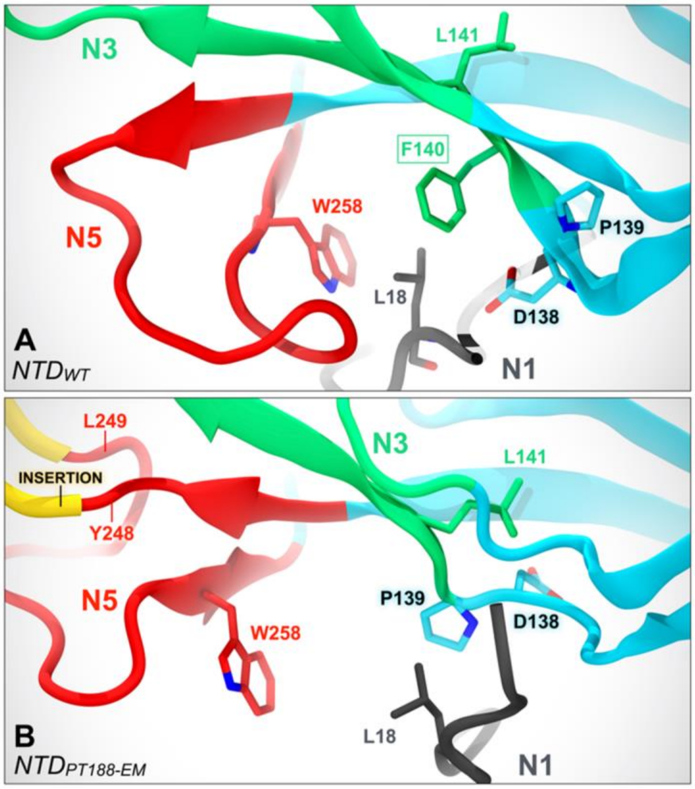

Figure S3. F140 deletion affects packing of N1, N3 and N5 loops. (A) Molecular representation of the NTD of the WT SARS-CoV-2 S protein as in the cryo-EM structure with PDB id 7JJI. F140 (N3) establishes hydrophobic contacts with L18 (N1) and W258 (N5). (B) Molecular representation of the model of the PT188-EM spike NTD after molecular dynamics simulations. The pattern of interactions is disrupted by the deletion of F140, leading to a loosening of the N1/N3/N5 loop packing.

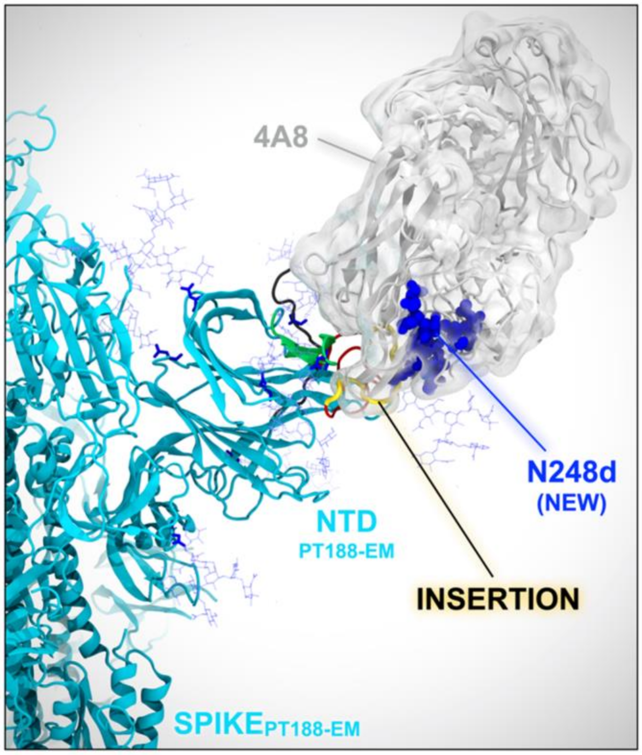

Figure S4. Insertion of 11 amino acids introduces a new N-glycan within N5 loop. (A) Molecular representation of the molecular dynamics-equilibrated model of the PT188-EM spike NTD based on PDB id 7C2L. The original cryo-EM structure used for this model already provided the coordinates for the NTD-bound 4A8 antibody (gray transparent surface). The relaxed model was aligned onto the cryo-EM coordinates, therefore retrieving the initial 4A8-bound pose, allowing for evaluation of steric compatibility.

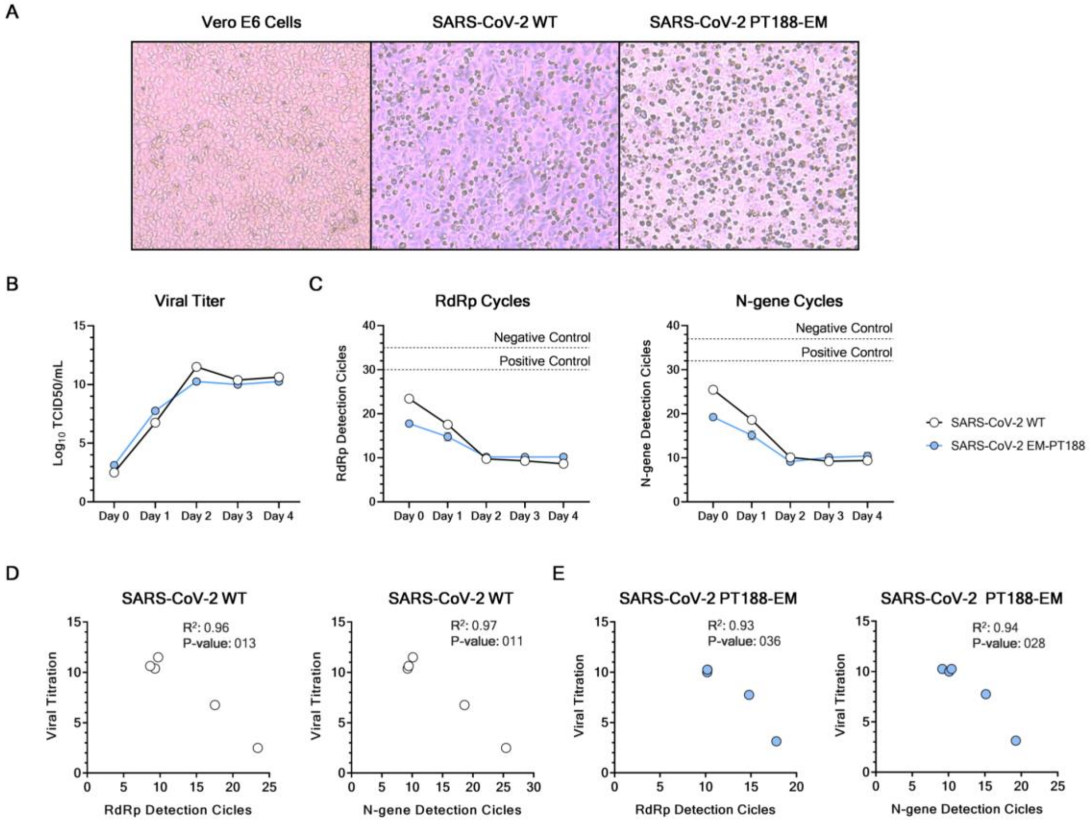

Figure S5. Evaluation of SARS-CoV-2 PT188-EM viral fitness. (A) Representative microscope images showing Vero E6 feeder cells alone and CPE observed for the SARS-CoV-2 WT and SARS-CoV-2 PT188-EM. (B) Viral titer of SARS-CoV-2 WT (white dots) and SARS-CoV-2 J08-EM (red dots) viruses shown as Log_{10} $TCID_{50}$/mL. (C) RT-PCR detection cycles for RdRp and N-gene. Dotted lines represent the negative and positive control thresholds per each gene. Curves show technical duplicates. (D – E) Correlation between viral titer and RdRp or N-gene detection cycles for SARS-CoV-2 WT (white dots) and SARS-CoV-2 PT188-EM (red dots) respectively.

Supplementary Table

Table S1. SARS-CoV-2 escape mutant summary. The table shows the starting dilution, passages, viral titer and neutralization activity of the plasma per each passage to generate the authentic virus SARS-CoV-2 escape mutant.

**Sample ID**	**Starting Dilution**	**Passage**	**Days of Incubation (Days per Passage)**	**Viral Titer**	**Mutation (% Virion Frequency)**	**Neutralization Titer**
**PT188**	1/10	0	5 (5)	$10^{5.4}$	-	640
**PT188**	1/10	1	10 (5)	$10^{5.4}$	-	640
**PT188**	1/10	2	15 (5)	$10^{5.7}$	-	640
**PT188**	1/10	3	20 (5)	$10^{5.7}$	-	640
**PT188**	1/10	4	25 (5)	$10^{5.2}$	-	640
**PT188**	1/10	5	31 (6)	$10^{5.1}$	-	640
**PT188**	1/10	6	38 (7)	$10^{4.9}$	-	640
**PT188**	1/10	7	45 (7)	$10^{4.2}$	Deletion F140 – NTD (39%)	320
**PT188**	1/10	8	53 (8)	$10^{4.3}$	Deletion F140 – NTD (100%)	160
**PT188**	1/10	9	59 (6)	$10^{4.4}$	-	160
**PT188**	1/10	10	66 (7)	$10^{4.5}$	-	160
**PT188**	1/10	11	73 (7)	$10^{4.0}$	-	160
**PT188**	1/10	12	80 (7)	$10^{3.9}$	Substitution E484K – RBD (100%)	40
**PT188**	1/10	13	85 (5)	$10^{5.7}$	Insertion $_{248a}$KTRNKSTSRRE$_{248k}$ - NTD (49%)	Not-neutralizing
**PT188**	1/10	14	93 (8)	$10^{5.4}$	Insertion $_{248a}$KTRNKSTSRRE$_{248k}$ - NTD (100%)	Not-neutralizing

SI References

1. Andreano E, *et al.* (2021) Extremely potent human monoclonal antibodies from COVID-19 convalescent patients. *Cell* 184(7):1821-1835.e1816.
2. Kundi M (1999) One-hit models for virus inactivation studies. *Antiviral research* 41(3):145-152.
3. Sali A & Blundell TL (1993) Comparative protein modelling by satisfaction of spatial restraints. *Journal of molecular biology* 234(3):779-815.
4. Watanabe Y, Allen JD, Wrapp D, McLellan JS, & Crispin M (2020) Site-specific glycan analysis of the SARS-CoV-2 spike. 369(6501):330-333.
5. Shajahan A, Supekar NT, Gleinich AS, & Azadi P (2020) Deducing the N- and O-glycosylation profile of the spike protein of novel coronavirus SARS-CoV-2. *Glycobiology* 30(12):981-988.
6. Casalino L, *et al.* (2020) Beyond Shielding: The Roles of Glycans in the SARS-CoV-2 Spike Protein. *ACS Central Science* 6(10):1722-1734.
7. Casalino L, *et al.* (2020) AI-Driven Multiscale Simulations Illuminate Mechanisms of SARS-CoV-2 Spike Dynamics. *bioRxiv : the preprint server for biology*.
8. Barros EP, *et al.* (2020) The Flexibility of ACE2 in the Context of SARS-CoV-2 Infection. *Biophysical journal*.
9. Yan R, *et al.* (2020) Structural basis for the recognition of SARS-CoV-2 by full-length human ACE2. 367(6485):1444-1448.
10. Humphrey W, Dalke A, & Schulten K (1996) VMD: visual molecular dynamics. *Journal of molecular graphics* 14(1):33-38, 27-38.
11. Olsson MHM, Søndergaard CR, Rostkowski M, & Jensen JH (2011) PROPKA3: Consistent Treatment of Internal and Surface Residues in Empirical pKa Predictions. *Journal of Chemical Theory and Computation* 7(2):525-537.
12. Phillips JC, *et al.* (2020) Scalable molecular dynamics on CPU and GPU architectures with NAMD. *The Journal of chemical physics* 153(4):044130.
13. Huang J, *et al.* (2017) CHARMM36m: an improved force field for folded and intrinsically disordered proteins. *Nature methods* 14(1):71-73.
14. Guvench O, Hatcher ER, Venable RM, Pastor RW, & Mackerell AD (2009) CHARMM Additive All-Atom Force Field for Glycosidic Linkages between Hexopyranoses. *J Chem Theory Comput* 5(9):2353-2370.
15. Huang J & MacKerell AD, Jr. (2013) CHARMM36 all-atom additive protein force field: validation based on comparison to NMR data. *Journal of computational chemistry* 34(25):2135-2145.
16. Ryckaert J-P, Ciccotti G, & Berendsen HJC (1977) Numerical integration of the cartesian equations of motion of a system with constraints: molecular dynamics of n-alkanes. *Journal of Computational Physics* 23(3):327-341.
17. Darden T, York D, & Pedersen L (1993) Particle mesh Ewald: An N·log(N) method for Ewald sums in large systems. 98(12):10089-10092.
18. Ermak DL & McCammon JA (1978) Brownian dynamics with hydrodynamic interactions. 69(4):1352-1360.
19. Martyna GJ, Tobias DJ, & Klein ML (1994) Constant pressure molecular dynamics algorithms. 101(5):4177-4189.

20. Feller SE, Zhang Y, Pastor RW, & Brooks BR (1995) Constant pressure molecular dynamics simulation: The Langevin piston method. 103(11):4613-4621.
